# The mealybug *Phenacoccus solenopsis* suppresses plant defense responses by manipulating JA-SA crosstalk

**DOI:** 10.1038/srep09354

**Published:** 2015-03-20

**Authors:** Peng-Jun Zhang, Fang Huang, Jin-Ming Zhang, Jia-Ning Wei, Yao-Bin Lu

**Affiliations:** 1 Zhejiang Provincial Key Laboratory of Biometrology and Inspection & Quarantine, College of Life Sciences, China Jiliang University, Hangzhou 310018, China; 2 State Key Laboratory Breeding Base for Zhejiang Sustainable Pest and Disease Control; Institute of Plant Protection and Microbiology, Zhejiang Academy of Agricultural Sciences, Hangzhou 310021, China; 3 State Key Laboratory of Integrated Management of Pest Insects & Rodents, Institute of Zoology, Chinese Academy of Sciences, Beijing 100080, China

## Abstract

Induced plant defenses against herbivores are modulated by jasmonic acid-, salicylic acid-, and ethylene-signaling pathways. Although there is evidence that some pathogens suppress plant defenses by interfering with the crosstalk between different signaling pathways, such evidence is scarce for herbivores. Here, we demonstrate that the mealybug *Phenacoccus solenopsis* suppresses the induced defenses in tomato. We found that exogenous JA, but not SA, significantly decreased mealybug feeding time and reduced nymphal performance. In addition, constitutive activation of JA signaling in *35s::prosys* plants reduced mealybug survival. These data indicate that the JA signaling pathway plays a key role in mediating the defense responses against *P. solenopsis*. We also found that mealybug feeding decreased JA production and JA-dependent defense gene expression, but increased SA accumulation and SA-dependent gene expression. In SA-deficient plants, mealybug feeding did not suppress but activated JA accumulation, indicating that the suppression of JA-regulated defenses depends on the SA signaling pathway. Mealybugs benefit from suppression of JA-regulated defenses by exhibiting enhanced nymphal performance. These findings confirm that *P. solenopsis* manipulates plants for its own benefits by modulating the JA-SA crosstalk and thereby suppressing induced defenses.

Plants possess an array of direct and indirect defenses to protect them against herbivore attack. The role of phytohormones and signaling pathways in the regulation of theses direct and indirect defenses is well established. The octadecanoid pathway, with the central phytohormone jasmonic acid (JA); the shikimate pathway, with the central phytohormone salicylic acid (SA); and the ethylene (ET) pathway are recognized as key signaling pathways in mediating plant defense responses^1^,^2^,^3^,^4^. According to the model proposed by Reymond and Farmer^5^, a plant tailors its defense responses to a specific attacker by eliciting signaling molecules from the three pathways to different degrees. Supporting evidence has been accumulating that the crosstalk between JA/ET and SA signaling pathways allows plants to fine-tune the induction of their defense in response to different herbivores or pathogens^6^,^7^,^8^,^9^. JA-SA crosstalk often results in reciprocal antagonism between these two pathways has been well-demonstrated to be an adaptive strategy that enhances plant fitness^10^.

Although crosstalk among different signaling pathways tailors plant responses to specific herbivores, the selective advantage of manipulating such crosstalk from the perspective of herbivores must be high. Some recent studies showed that insect herbivores can suppress induced defenses of plants^11^,^12^,^13^,^14^. In these cases, the suppression of plant defense responses is often associated with changes of phytohormone biosynthesis or signaling pathways. For example, oral secretions of the beet armyworm *Spodoptera exigua* can suppress JA and ET accumulation but enhance SA accumulation in *Nicotiana attenuata*^8^, and the SA induction in *Arabidopsis* facilitates the growth of *S. exigua* larvae^15^. Similarly, the silverleaf whitefly *Bemisia tabaci* activates SA-dependent responses and represses JA-dependent defense responses to their own advantage in *Arabidopsis thaliana*^11^,^16^,^17^. Considering the well-demonstrated JA-SA crosstalk, researchers speculated that insects can manipulate plant defenses for their own benefits by modulating the JA-SA crosstalk^18^. Although this speculation has been supported by a few studies^12^,^13^,^15^,^19^, the mechanisms involved have received little attention.

Recently, we found that the solenopsis mealybug *Phenacoccus solenopsis*, which is a newly recognized invasive insect in China^20^, can suppress the induction of JA-regulated genes and defense metabolites to enhance its nymphal performance on cotton^21^. However, the underlying mechanism involved in the suppression of JA defense responses by *P. solenopsis* remains unknown. Considering that *P. solenopsis* may also activate the induction of SA-dependent responses and show enhanced performance on SA-treated plants^21^, we hypothesized that suppression of JA defense by *P. solenopsis* might be mediated by crosstalk with the SA signaling pathway. To test this hypothesis, we used wild-type tomato that is a host plant for *P. solenopsis* and two transgenic tomato lines including *NahG* with defective SA biosynthesis and *35S::prosys* with constitutive JA signaling. First, we examined the effects of exogenous JA, SA, and herbivory treatments on the performance of *P. solenopsis* larvae. Second, we used the electronic penetration graph (EPG) technique to record the *P. solenopsis* feeding behavior on undamaged plants and plants treated with JA, SA, and herbivory. Third, we quantified the accumulation of endogenous JA and SA as well as the transcript levels of JA- and SA-dependent genes in host plants in response to *P. solenopsis* feeding. Finally, we examined the survival rate of *P. solenopsis* on *NahG*, *35S::prosys*, and their corresponding wild-type plants, and quantified JA and SA in the two transgenic lines in response to *P. solenopsis* feeding. Our results demonstrate that *P. solenopsis* feeding enhances SA accumulation, which suppresses the JA signaling pathway. As a consequence of this herbivore modification of JA-SA crosstalk, the performance of *P. solenopsis* nymphs is enhanced.

## Methods

### Plants and insects

Wild-type tomato (*Lycopersicon esculentum*) cv Moneymaker (MM) is the parental line for the SA-non-accumulating *NahG* mutant. Wild-type tomato (*Solanum lycopersicum*) cv Castlemart (CM) is the parental line for the transgenic tomato line *35s::prosys* in which JA signaling is constitutive. *35S::prosys* seeds were collected from a *35S::prosys* homozygote that had been backcrossed five times to its wild-type line cv Castlemart^22^,^34^. Tomato seedlings were grown in 500-ml pots containing a commercial potting mix (Fafard Growing Mix 1, Agawam, MA), and were kept in an insect-free greenhouse compartment under natural light and 28/24°C. Plants with four to five fully expanded leaves were used for experiments.

The mealybug, *Phenacoccus solenopsis* Tinsley (Hemiptera: Pseudococcidae), was originally collected from *Hibiscus rosa-sinensis* in Hangzhou (30°10′N, 120°15′E), China, and was maintained on wild-type tomato cv Moneymaker in a climate-controlled room (25 ± 3°C, 60–70% RH, 12L: 12D photoperiod).

### Plant treatments

1. Chemical treatment: Jasmonic acid or salicylic acid (Sigma-Aldrich) was dissolved in 0.5 mL of acetone and dispersed in water (containing 0.1% Tween 20) to produce a 1.0 mM JA or SA solution. Each plant was sprayed with 1.0 mL/leaf of the JA or SA solution with a hand-sprayer. Twenty-four hours later, JA- or SA-treated plants were used for experiments.
2. Mealybug treatment: A mixture of third-instar nymphs and newly emerged adults (40 in total) of *P. solenopsis* were carefully transferred onto each plant and allowed to feed freely for 6, 12, 24, 72, or 120 h. After that, leaf samples were collected for phytohormone analysis. Plants pre-infested with 40 *P. solenopsis* adults (2- to 4-days old) for 120 h were used for performance bioassays, the EPG experiment, and gene-expression analysis.
3. Control treatment: In a preliminary experiment, we found that treatment with a water solution (including 0.5 mL of acetone and 0.1% Tween 20) had no effect on the performance of *P. solenopsis* reared on tomato plants. Thus, healthy and intact plants that received no treatment were used as control plants.

To avoid the potential interference among plants from different treatments, plants from the same treatment were maintained in a separate climate-controlled room (25 ± 3°C, 60–70% RH, 12L: 12D photoperiod). And each plant was individually kept in a glass cage (25 × 25 × 50 cm).

### Performance of mealybugs feeding on tomato plants

We determined the effects of JA, SA, and herbivory treatments on the survival of *P. solenopsis*. Young nymphs (≤24 h old) from the same cohort were transferred onto the leaves (50 nymphs per plant) of control plants or plants treated with JA, SA, or herbivory (mealybugs). The plants were kept in a climate-controlled room (26 ± 2°C, 60–70% RH, 12L: 12D photoperiod). The mealybugs were assessed twice daily for survival, and survival rates were calculated at 14, 21, and 28 d after the young nymphs had been placed on the plants. Each treatment was represented by four replicate plants.

A similar experiment was performed to determine the effects of control, JA, SA, and herbivory treatments on *P. solenopsis* developmental duration (from egg to last molt). The procedure was the same as that described in the previous paragraph except that developmental duration was recorded twice daily until adults emerged, and each treatment was represented by five replicate plants.

### EPG recording of P. solenopsis feeding behavior on tomato plants

*P. solenopsis* stylet penetration activities on the leaves of control plants or plants treated with JA, SA, or herbivory (mealybugs) were recorded using a four-channel DC-EPG system (Giga-4; Wageningen, The Netherlands). The method used for recording was the same as that described by Huang *et al.* (2014)^23^. In brief, 7- to 8-day-old adult females were carefully collected from tomato plants. The dorsal wax was partially and gently removed from each specimen with a fine brush to enable the attachment of a gold wire electrode (18 μm diameter, 2 cm long). Experiments were conducted in a Faraday cage in the laboratory at 27 ± 2°C. The recordings were started around 08:30 hours and continued for 12 h under florescent light. For each treatment, 20 recordings, each done with a separate mealybug on a separate plant, were used for characterization and analysis of the EPG signals using the EPG analysis worksheet.

### Quantification of endogenous JA and SA

Endogenous JA and SA in tomato plants were quantified as described by Wei *et al.* (2013)^24^ and Almeida-Trapp *et al.* (2014)^25^. In brief, plant material (250–300 mg) was frozen and ground in liquid nitrogen. The resulting powder was mixed with 4 mL of HPLC grade methanol (Sigma-Aldrich) and kept at −20°C for 12 h. For quantification, [9, 10]-dihydro-JA (300 ng) and d6-SA (500 ng) were added as internal standards. JA, SA, and their internal standards were partitioned to an aqueous phase by centrifugation and vaporization. After three rounds of freezing and thawing, the aqueous phase was centrifuged, and the pH of the supernatant was adjusted to 3.0 using 0.1 M HCl. JA, SA, and their internal standards were extracted from the supernatant with an equal volume of ethyl acetate and then dried. The dried extract was re-suspended in 0.1 M acetic acid and loaded onto a C18 column (Waters Company, Milford, MA, USA). The C18 column was sequentially eluted with a series of solvent mixtures [acetic acid/methanol (v/v) at 83/17, 60/40 and 40/60]. After evaporation of the solvent and esterification of the residue using excess ethereal diazomethane, the elution sample volume was adjusted to 50 mL with ethyl acetate. Samples were analyzed using a GC/MS system (6890N/5973 MSD, Agilent Technologies, Inc., Palo Alto, CA, USA) equipped with an HP-5-MS column (30 m × 0.25 mm × 0.25 mm; 19091S-433, J&W Scientific, Agilent Technologies). Endogenous JA, SA, and their internal standards were analyzed in full-scan mode as described by Wei *et al.* (2013)^24^.

### Quantitative real-time PCR

Total RNA was extracted and purified as described by Zhang *et al.* (2013)^19^. First stand cDNA was synthesized from 200 ng RNA using a First-Strand cDNA Synthesis Kit (Bio-Rad, Hercules, CA, USA) according to the manufacturer's instructions. To quantify *LoxA*, *Pin2*, *PR1*, and *GluA* transcript levels in different samples, real-time quantitative RT-PCR was performed. The real-time PCR was carried out on an ABI 7500 Real Time PCR System (Applied Biosystems, Foster City, CA) with a 96-well rotor. The amplification reactions were performed in a 20-μL final volume containing 10 μl of iQ^TM^ SYBR® supermix (BioRad, Hercules, CA), 0.8 μL of forward primer (5 μM) and reverse primer (5 μM) pairs, and 2 μL of cDNA first-strand template. Thermal cycling conditions were 5 min at 95°C followed by 40 cycles of 15 s at 95°C and 15 s at 58°C. The gene-specific primers, which were designed and checked as described previously^29^, were as follows: for *LoxA* (U09026; F: 5′- TGGTAGACCACCAACACGAA-3′, R: 5′-GACCAAAACGCTCGTCTCTC-3′); for *Pin-2* (AY129402; F: 5′-TGATGCCAAGGCTTGTACTAGAGA-3′, R: 5′-AGCGGACTTCCTTCTGAACGT-3′); for *PR-1a* (M69247; F: 5′-GAGGGCAGCCGTGCAA-3′, R: 5′-CACATTTTTCCACCAACACATTG-3′); for *GluA* (M80604; F: 5′-TCA GCAGGGTTGCAAAATCA-3, R: 5′-CTCTAGGTGGGTAGGTGTTGGTTAA-3′); and for *GAPDH* (U93208; F: 5′- CTCCATCACAGCCACTCAGA-3′, R: 5′-TTCCACCTCTCCAATCCTTG-3′). All reactions were run in duplicate, and average values were used in the analyses. Normalized gene expression was calculated using the 2^−ΔCt^ method with *GAPDH* as the endogenous control gene, and values were subsequently log_2_ transformed.

### Statistical analysis

Fisher's protected least significant difference (PLSD) test of ANOVA was used to analyze phytohormone data and the data for development time and survival rate. The gene expression data were statistically analyzed by one-way ANOVA. Proportional data were arcsine square root transformed for analysis and back-transformed to percentages for presentation. EPG data were transformed where appropriate (square-root transformation for number of occurrences and natural log transformation for duration) and analyzed by one-way ANOVA.

## Results

### Performance of *P. solenopsis* on tomato plants

**Figure 1:**
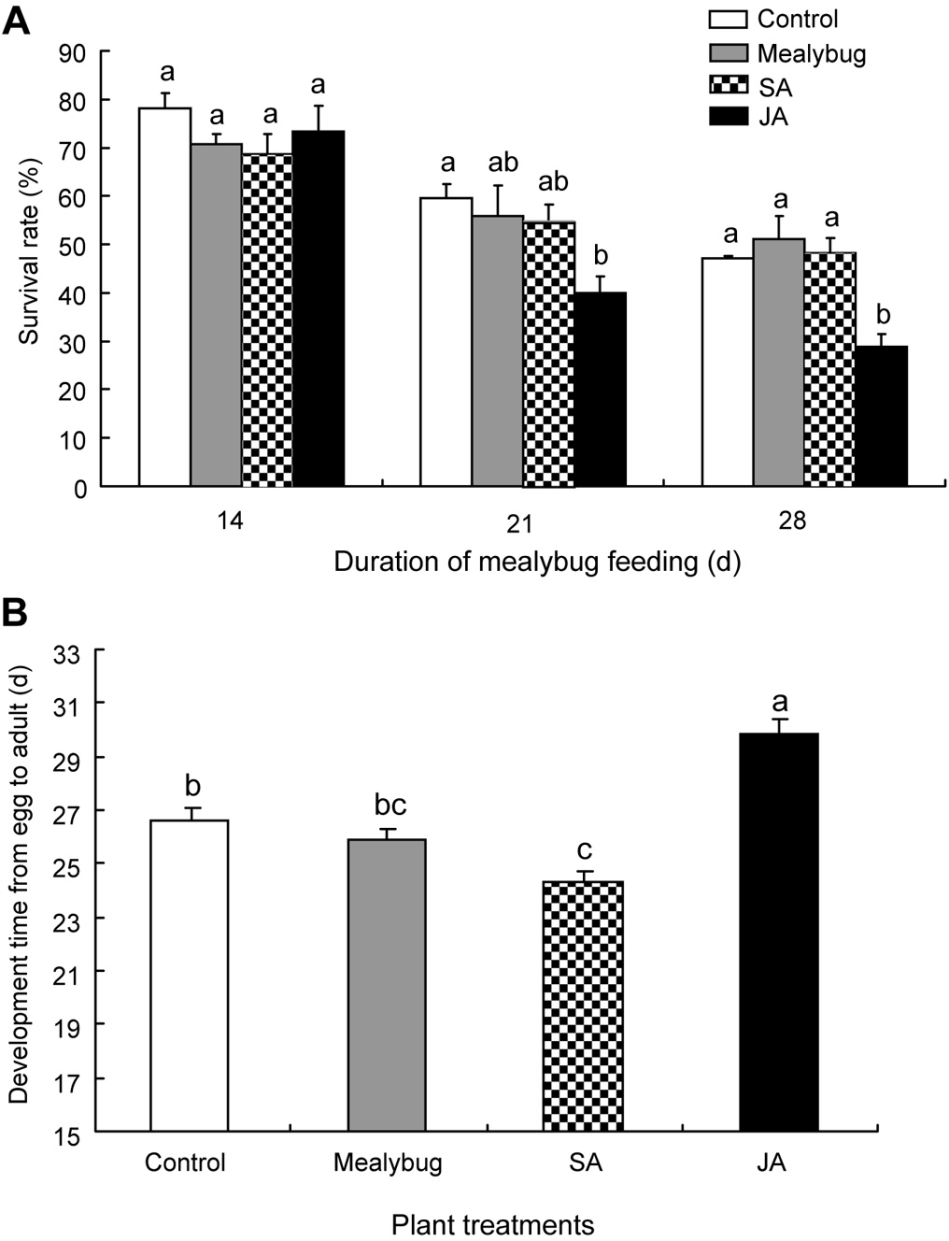
Performance of *P. solenopsis* on non-infested (Control), *P. solenopsis*-infested (Mealybug), SA-treated, and JA-treated tomato plants. (A) Survival rate of *P. solenopsis* at different time points, and (B) developmental duration from egg to adult of *P. solenopsis*. Values are means ± SE, n = 4 in (A) and 25–33 in (B). For each time point in (A) and for the four treatments in (B), bars with different letters are significantly different (Fisher's PLSD test of ANOVA; *P* < 0.05).

After 14 d of infestation, the survival rate of *P. solenopsis* nymphs on control plants was 78.3 ± 3.3%, and survival on JA-, SA-treated, or *P. solenopsis*-infested plants did not significantly differ from survival on control plants (*F*_3, 12_ = 1.14, *P* = 0.37; Fig. 1A). In contrast, survival at 21 d significantly differed among the four treatments (*F*_3, 12_ = 3.73, *P* = 0.04; Fig. 1A). At 21 d after infestation, survival was significantly lower (*P* = 0.04) on JA-treated plants than on control plants; survival did not differ on SA-treated, *P. solenopsis*-infested, and control plants (SA: *P* = 0.86; mealybug: *P* = 0.94; Fig. 1A). Survival at 28 d also differed among the four treatments (*F*_3, 12_ = 10.39, *P* = 0.001; Fig. 1A). At 28 d after infestation, survival was significantly lower (*P* = 0.007) on JA-treated plants than on control plants, but survival did not significantly differ on SA-treated, *P. solenopsis*-infested, and control plants (SA: *P* = 0.99; mealybug: *P* = 0.81; Fig. 1A).

Developmental duration of *P. solenopsis* from egg to adult significantly differed among the four treatments (*F*_3, 109_ = 22.87, *P* < 0.001; Fig. 1B). Developmental duration was significantly longer on JA-treated plants than on control plants (*P* < 0.001; Fig. 1B) but was significantly shorter on SA-treated plants than on control plants (*P* = 0.01; Fig. 1B). Pre-infestation by *P. solenopsis* did not affect the developmental duration of *P. solenopsis* (*P* = 0.74; Fig. 1B).

### Feeding behavior of *P. solenopsis* as indicated by EPG

EPG detects the electrical fluctuations caused by probing behavior of mealybugs during foraging and feeding^26^,^27^. Three main phases during stylet penetration have been defined: the pathway phase, the xylem phase, and the phloem (or sieve element) phase^28^. Given that the probing behavior of *P. solenopsis* has seldom been recorded in the xylem phase^23^,^27^, we mainly analyzed the occurrence of cell puncture (as indicated by the rate at which potential dropped) in the pathway phase and the duration and insertion rate in the phloem (or sieve element) phase. Based on the EPG data, the mealybug treatment did not affect the feeding behavior of *P. solenopsis* adults (Table 1). The JA treatment significantly reduced the total feeding time in the phloem phase but did not affect the feeding rate in the phloem phase, the number of probes, or the rate of cell puncture (Table 1). The SA treatment significantly increased the rates of feeding in the phloem phase and cell puncture in the pathway phase, but did not affect the total feeding time in the phloem phase or the number of probes (Table 1).

### Quantification of endogenous JA and SA in host leaves

**Figure 2:**
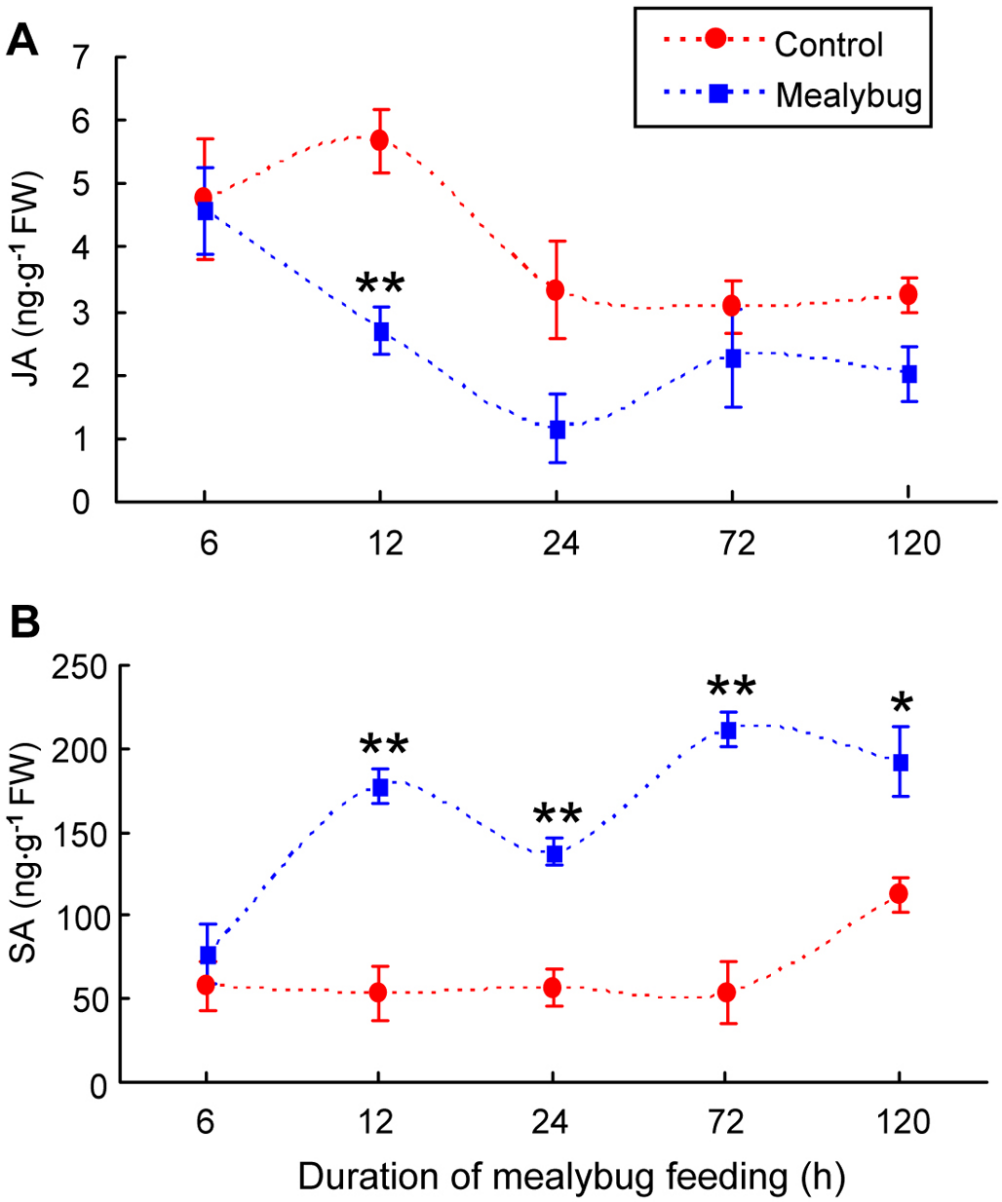
The accumulation of (A) JA and (B) SA in non-infested tomato leaves (Control) and in *P. solenopsis*-infested (Mealybug) tomato leaves at different time points. Values are means ± SE of three biological replicates. Asterisks above bars indicated significant differences compared to the control (Fisher's PLSD test of ANOVA; * P < 0.05; ** P < 0.01). FW means fresh weight.

The amount of JA was significantly lower in *P. solenopsis*-infested leaves than in control leaves at 12 h after infestation (*P* = 0.008) but not at 6, 24, 72, or 120 h after infestation (6 h: *P* = 0.88; 24 h: *P* = 0.08; 72 h: *P* = 0.42; 120 h: *P* = 0.08) (Fig. 2A). This is consistent with previous findings that the effect of herbivory on endogenous JA level generally occurs within 12 h after infestation^8^,^29^.

The amount of SA was significantly higher in *P. solenopsis*-infested leaves than in control leaves at 12, 24, 72, and 120 h after infestation (12 h: *P* = 0.003; 24 h: *P* = 0.004; 72 h: *P* = 0.002; 120 h: *P* = 0.03), but not at 6 h after infestation (*P* = 0.45; Fig. 2B).

**Figure 3:**
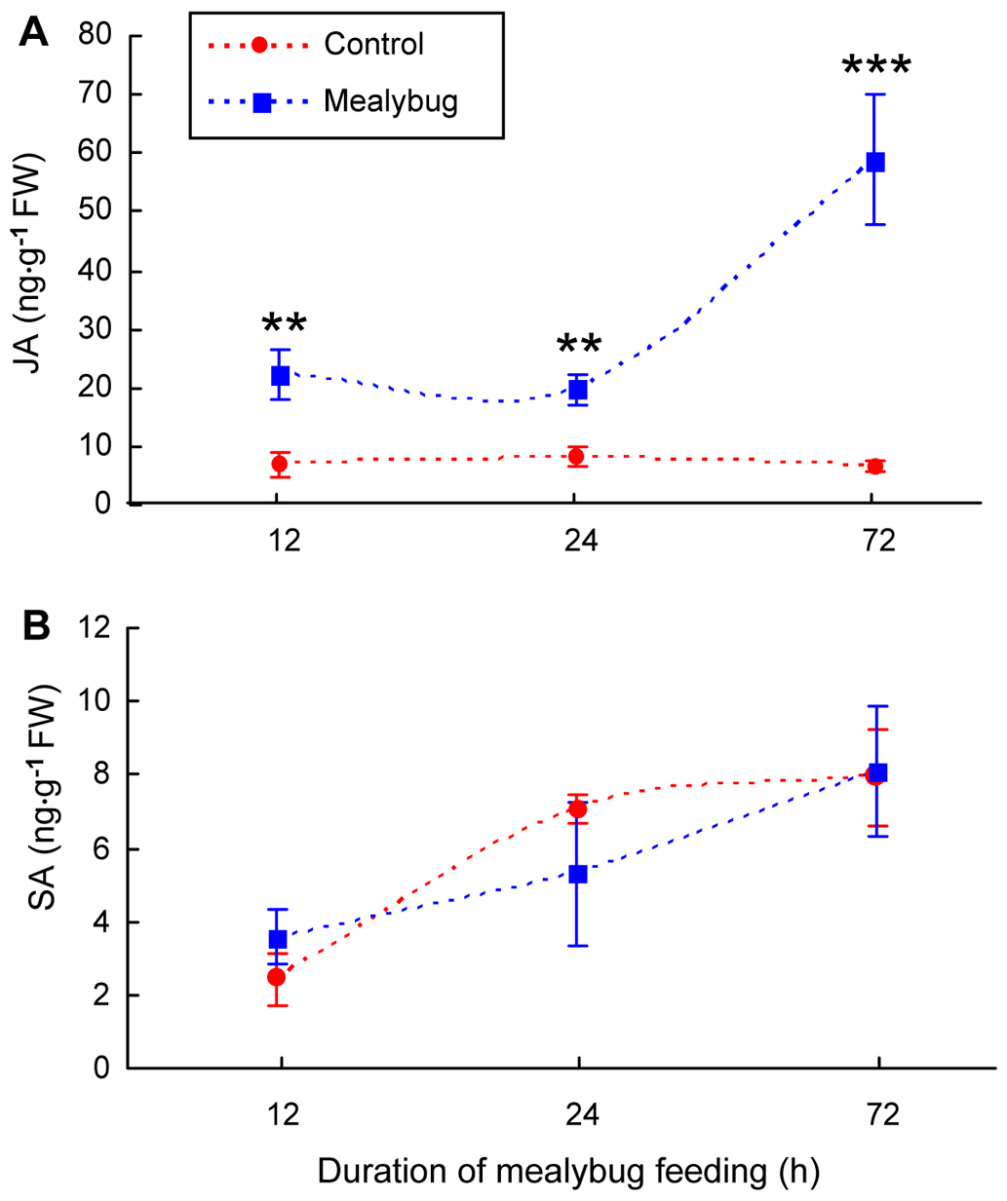
Accumulations of (A) JA and (B) SA in non-infested leaves (Control) and in *P. solenopsis*-infested (Mealybug) leaves of *NahG* tomato plants. Values are means ± SE of three biological replicates. Asterisks above bars indicated significant differences compared to the control (Fisher's PLSD test of ANOVA; ** P < 0.01; *** P < 0.001). FW means fresh weight.

To test whether SA signaling plays a key role in mediating the suppression of JA defensive responses by *P. solenopsis*, we further examined the JA and SA levels in *NahG* (SA-deficient) and *35s::prosys* (JA-overexpression) plants infested with *P. solenopsis*. For *NahG* plants, the amount of JA was significantly higher in infested leaves than in control leaves at 12, 24, and 72 h after infestation (12 h: *P* = 0.004; 24 h: *P* = 0.002; 72 h: *P* = 0.001) (Fig. 3A). In contrast, the amounts of SA in control *NahG* plants were consistently low (<9 ng g^−1^ fresh weight), and *P. solenopsis* infestation of *NahG* plants did not increase SA amounts at 12, 24, and 72 h after infestation (12 h: *P* = 0.12; 24 h: *P* = 0.20; 72 h: *P* = 0.91) (Fig. 3B).

**Figure 4:**
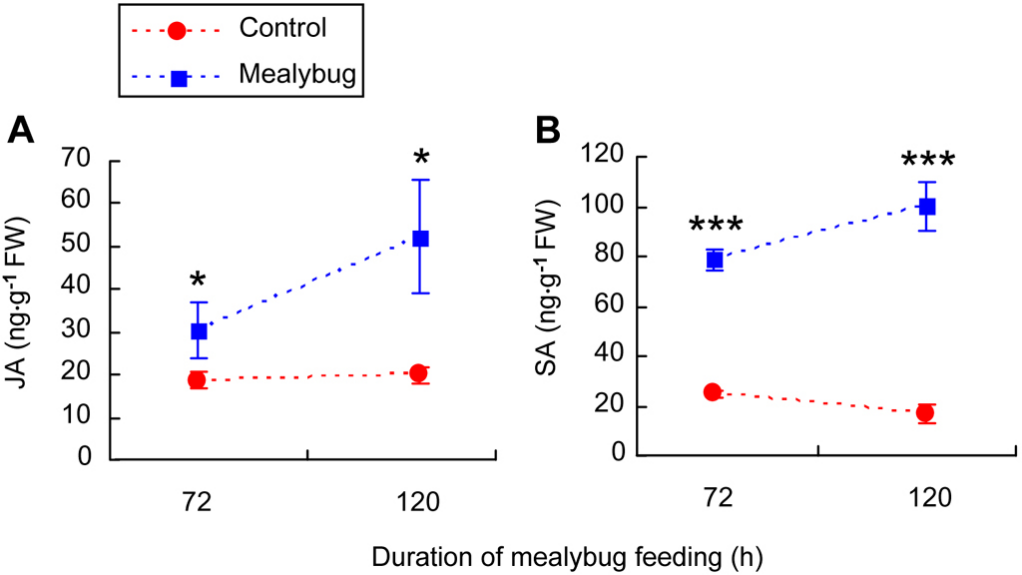
Accumulations of (A) JA and (B) SA in non-infested leaves (Control) and in *P. solenopsis*-infested (Mealybug) leaves of *35s::prosys* tomato plants. Values are means ± SE of three biological replicates. Asterisks above bars indicated significant differences compared to the control (Fisher's PLSD test of ANOVA; * P < 0.05; *** P < 0.001). FW means fresh weight.

For *35s::prosys* plants, the amounts of JA were significantly higher in infested leaves than in control leaves at 72 and 120 h after infestation (72 h: *P* = 0.04; 120 h: *P* = 0.015) (Fig. 4A). Similarly, the amounts of SA were significantly higher in infested leaves than in control leaves at 72 and 120 h after infestation (72 h: *P* = 0.001; 120 h: *P* = 0.001) (Fig. 4B).

### Changes in gene-expression in response to *P. solenopsis* feeding

**Figure 5:**
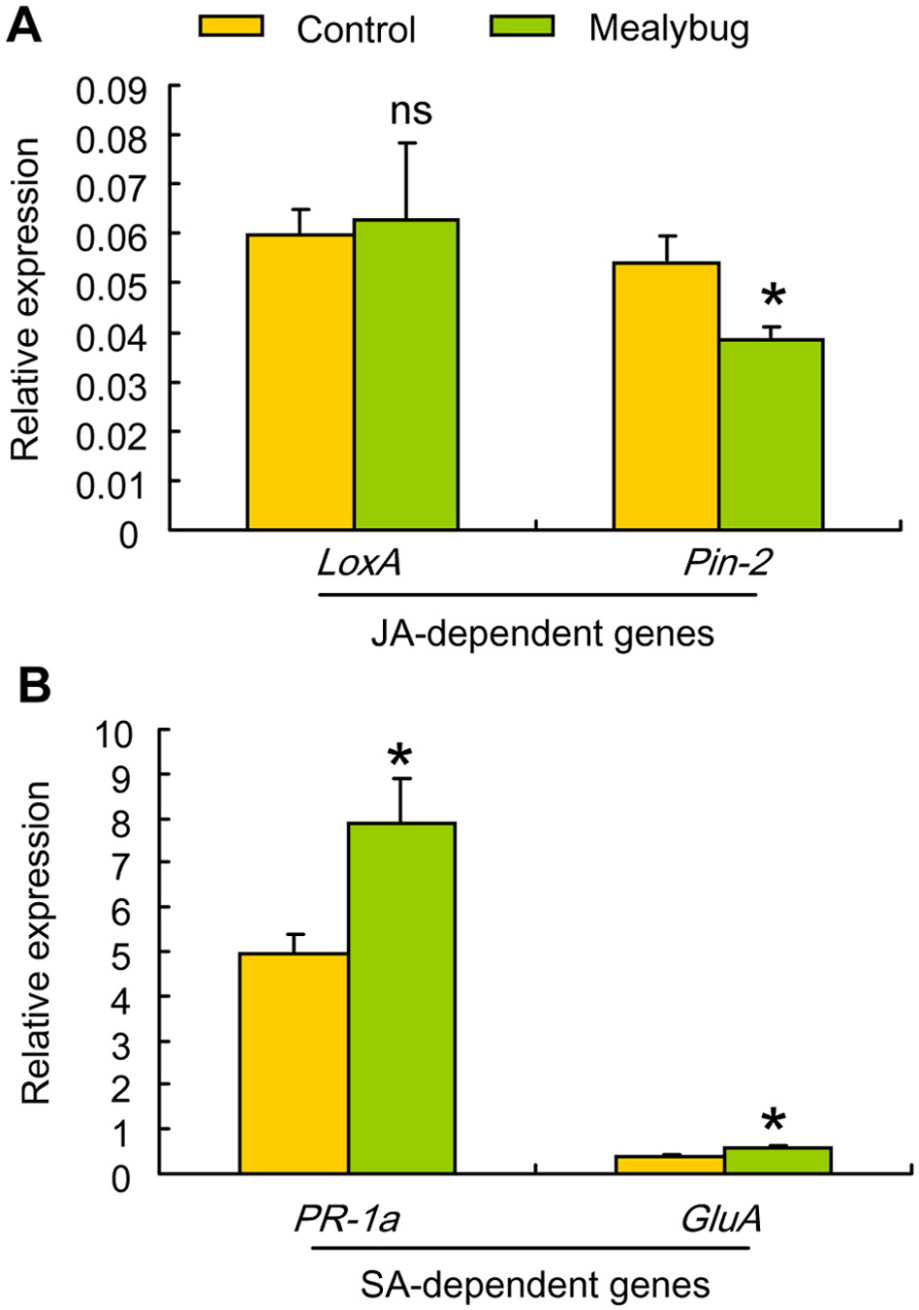
Expression of (A) JA-dependent genes and (B) SA-dependent genes in non-infested tomato leaves (Control) and in *P. solenopsis*-infested (Mealybug) tomato leaves. Wild-type tomato (Moneymaker) plants were infested for 120 h with 40 adults of *P. solenopsis*. Expression was measured by real-time PCR. Values are means ± SE of three biological replicates. Asterisks above bars indicated significant differences compared to the control (one-way ANOVA; ns, not significant; * P < 0.05).

To further investigate the effects of *P. solenopsis* feeding on JA- or SA-dependent defense responses, we examined the transcript levels of two JA-dependent genes (*LoxA* and *Pin-2*) and SA-dependent genes (*PR-1a* and *GluA*). Lipoxygenase (*LOX*) is a key enzyme in the biosynthesis of JA along the octadecanoid pathway^30^. Proteinase inhibitor II (*Pin-2*), a serine proteinase inhibitor with trypsin and chymotrypsin inhibitory activities, is known to confer insect resistance in many Solanaceae plants^31^. In tomato, *LoxA* and *Pin-2* are marker genes of the JA signaling pathway^33^. *PR-1a* and *GluA* are known as two pathogenesis-related genes, which are mainly regulated by the SA signaling pathway^32^,^33^. Mealybug feeding significantly suppressed the expression of *Pin-2* (*F*_1, 5_ = 8.47, *P* = 0.04) but not of *LoxA* (Fig. 5A). In contrast, mealybug feeding significantly induced the expression of *PR-1a* and *GluA* (*PR-1a*: *F*_1, 5_ = 9.17, *P* = 0.04; *GluA*: *F*_1, 5_ = 12.32, *P* = 0.02) (Fig. 5B).

### Performance of *P. solenopsis* on tomato mutants

**Figure 6:**
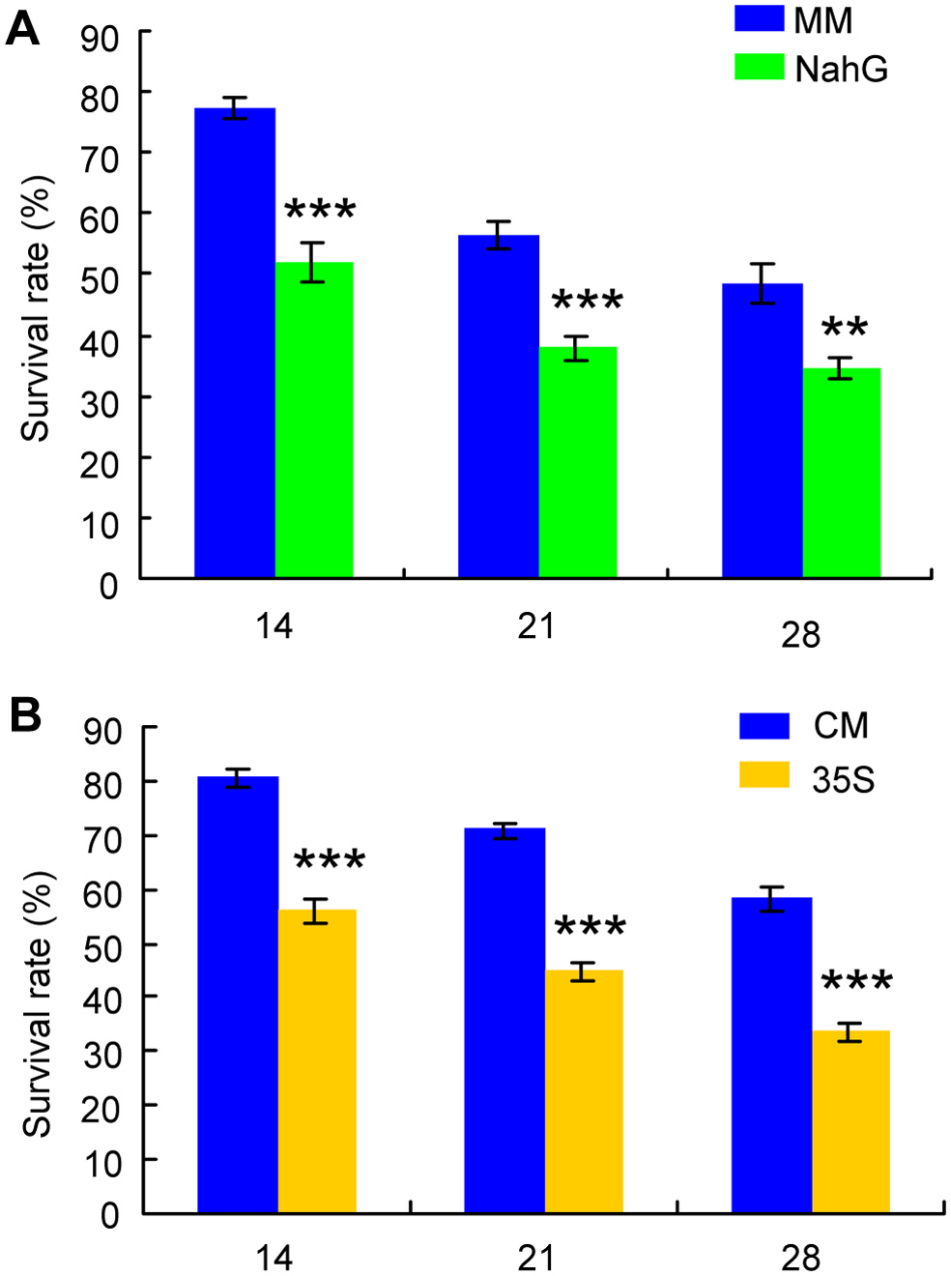
Survival rate of *P. solenopsis* on (A) MM (wild-type cv Moneymaker) vs. *NahG* tomato plants and on (B) CM (wild-type cv Castlemart) vs. *35s::prosys* tomato plants. Values are means ± SE (n = 4). Asterisks above bars indicated significant differences compared to the wild type (Fisher's PLSD test of ANOVA; ** P < 0.01; *** P < 0.001).

Survival of *P. solenopsis* were significantly lower on SA-deficient *NahG* plants than on wild-type MM plants at 14, 21, and 28 d after infestation (14 d: *P* < 0.001; 21 d: *P* < 0.001; 28 d: *P* = 0.008; Fig. 6A). Likewise, survival of *P. solenopsis* was significantly lower on JA-overexpression *35s::prosys* plants than on wild-type CM plants at 14, 21, and 28 d after infestation (14 d: *P* < 0.001; 21 d: *P* < 0.001; 28 d: *P* < 0.001) (Fig. 6B).

## Discussion

The results of the present study show that application of JA but not SA significantly decreases the feeding time of the mealybug *P. solenopsis* in the phloem tissue and consequently decreases nymphal performance. In addition, constitutive expression of JA signaling in *35s::prosys* plants reduces *P. solenopsis* survival. These data demonstrate that the JA signaling pathway plays a key role in mediating the defense responses against *P. solenopsis* in tomato. This result is consistent with a previous finding that the JA-dependent defense pathway may be involved in basal defense against *P. solenopsis* in cotton^21^. Our data further show that *P. solenopsis* can suppress the JA-dependent responses, as evidenced by the decrease in expression of JA-dependent *Pin2* and the reduction of JA level in infested plants. Meanwhile, *P. solenopsis* can induce the SA-dependent responses as indicated by the SA accumulation and increased expression of SA-dependent *PR-1a* and *GluA* in infested plants. That the suppression of JA defense responses is mediated by the SA signaling pathway is indicated by the accumulation of JA in infested SA-deficient *NahG* plants and significantly higher levels of SA in infested WT plants. Moreover, *P. solenopsis* survival is better on control WT plants than on SA-deficient *NahG* plants and on *35s::prosys* plants that constitutively express JA, which suggests that manipulation of the pathways by nymphs allows them to perform better. Taken together, our results indicate that *P. solenopsis* can manipulate plants for its own benefit by modulating JA-SA crosstalk in order to suppress induced defenses.

The JA signaling pathway, including the wound hormones JA and JA-Ile, is widely recognized as a key regulator of plant defense against insect herbivores^1^,^34^,^35^. Several recent studies have found that insects can suppress JA-mediated defense responses to enhance their performance on food plant^11^,^12^,^17^,^19^. These suppression mechanisms often involve some herbivore effectors, including specific enzymes from oral secretions^36^, egg-derived elicitor^12^, and vectored microorganisms^14^,^37^. For example, caterpillars of the beet armyworm *Spodoptera exigua* can suppress the accumulation of JA and the induction of JA-dependent genes in *Arabidopsis* by releasing effectors in oral secretions^38^. Glucose oxidase in the saliva of *Helicoverpa zea* caterpillars was the first effector identified that reduces JA-regulated nicotine production in *Nicotiana tabacum*^36^. Similarly, phloem-feeding insects like aphids and whiteflies can inhibit the JA defense responses in different plant species although the effectors involved have not been identified^11^,^39^,^40^. However, the mechanism by which plants respond to herbivore effectors and eventually mediate the suppression of JA signaling has not been extensively studied.

Considering that pathogens can reduce plant defenses by manipulating antagonistic crosstalk between JA and SA signaling pathways^41^, researchers have speculated that insects can circumvent plant defenses in a similar way^18^. This possibility has been supported by several studies. For instance, oviposition by the cabbage butterfly *Pieris brassicae* induces SA accumulation and reduces the induction of JA-regulated defense genes in *Arabidopsis* and leads to the enhanced performance of *Spodoptera littoralis*; that the suppression of JA-dependent genes and enhancement of *S. littoralis* performance were not observed in the SA-deficient mutant *sid2-1* indicates that SA mediates this phenomenon^12^. In tomato, larvae of Colorado potato beetle, *Leptinotarsa decemlineata*, use bacteria in their oral secretions to decrease JA accumulation, decrease JA-responsive anti-herbivore defenses, and increase SA production; these effects were not observed, however, in SA-deficient plants^13^. In the present study, we found that the expression level of the JA-dependent gene *Pin-2* and JA production were significantly lower in *P. solenopsis*-infested plants than in non-infested plants, which indicates that, unlike herbivores in the two above cases, *P. solenopsis* does not prevent induction of JA-regulated defenses but reduces the constitutive level of JA defenses. This is consistent with the previous finding that *P. solenopsis* feeding strongly reduces the constitutive level of JA-regulated gossypol in cotton^21^. Our data further showed that if SA signaling is blocked, *P. solenopsis* does activate JA accumulation. Similar phenomena were also observed in the interaction between the whitefly *Bemisia tabaci* and *Arabidopsis*. The expression level of the JA-regulated defense genes *PDF1.2* and *VSP1* is significantly lower in *B. tabaci*-infested plants than in non-infested plants, indicating that *B. tabaci* reduces the constitutive levels of defense genes; however, once SA synthesis or its action is impaired, *B. tabaci* feeding does activate the induction of the two genes^11^,^19^. Considering that chewing insects cause extensive tissue damage whereas phloem-feeding insects cause minimal tissue damage^18^, the above cases indicated that the mode of mechanical damage caused by different herbivores possibly might be an additional factor that influences the strength of the reciprocal antagonism between JA and SA pathways. Collectively, these data indicate that herbivore modulation of the antagonistic crosstalk between the JA and SA signaling pathways might be more intricate than previously thought^10^.

Our results showed that *P. solenopsis* feeding did not affect the expression level of *LoxA*, which encodes a key enzyme involved in JA biosynthesis^30^, but decreased the expression level of JA-dependent *Pin-2* and endogenous JA level. Based on these data we speculated that the suppression of JA defense responses by *P. solenopsis* is likely located downstream of JA biosynthesis pathway. This speculation is supported by the observation that *B. tabaci* nymphs induce the upstream JA-responsive genes (*LOX2* and *OPR3*), but suppress the downstream JA-responsive gene (*VPS1* and *PDF1.2*) in *Arabidopsis*^11^,^19^, indicating that nymph feeding can suppress the downstream components of the JA signaling pathway. Future experiments should attempt to explore the target genes manipulated by phloem-feeding insects in the JA signaling pathway.

In addition to the role of SA in the suppression of JA defense responses, the consistent accumulation of SA induced by *P. solenopsis* feeding might benefit the development of larvae. One reason is that exogenous SA treatment strongly increases the feeding rate of *P. solenopsis* in the phloem tissue (Table 1), which might directly contribute to rapid larval development (Fig 1B). Alternatively, given that SA pathway plays a central role in plant defense against microbial and fungal pathogens^6^, the accumulation of SA near the feeding site might protect the leaf tissue from infection and ensure that the tissue retains its full nutrient content (i.e., is not degraded) during larval feeding.

Although considerable SA accumulated in response to *P. solenopsis* infestation, JA accumulation was also strongly induced by *P. solenopsis* infestation of the *35s::prosys* plant. Because JA signaling is constitutively enhanced in the *35s::prosys* plant^22^, we speculate that if the JA signaling is activated before SA signaling, the tomato plant might become insensitive to SA-mediated suppression of JA signaling by mealybug infestation. Our speculation is supported by a previous study in which simultaneous activation of the JA and ET signaling before induction of the SA response rendered *Arabidopsis* plants insensitive to SA-mediated suppression of JA-responsive gene expression^42^. These findings support the view that the sequence of hormone activation affects realization of the antagonism between JA and SA crosstalk^7^,^43^. Alternatively, we cannot rule out the possibility that the enhanced SA level induced by mealybug feeding is insufficient to suppress JA accumulation in the *35s::prosys* plant.

The mealybug *P. solenopsis* has been rapidly spreading throughout South China, where it is causing serious economic losses to cotton production^20^. Our data confirm that, at least in the laboratory, *P. solenopsis* can circumvent plant defenses by modulating the JA-SA crosstalk. This capability has possibly facilitated the rapid invasion of mealybugs in China and elsewhere^21^. Future studies should determine whether the modulation of the JA-SA antagonism by *P. solenopsis* occurs in the field, and if so, whether it enhances *P. solenopsis* population development.

## Author Contributions

P.J.Z. and Y.B.L. conceived and designed the research. P.J.Z., F.H., J.M.Z. and J.N.W. conducted the experiments and analyzed the data. F.H. and P.J.Z. interpreted the results and wrote the paper.

## Figures and Tables

**Table 1:** Feeding behavior of the mealybug *P. solenopsis* as indicated by EPG recordings

Plant Treatments	Feeding parameters							
	Total duration of SEP feeding (min)	P value	Rate of SEP feeding	P value	No. of probes	P value	Rate of potential drop	P value
Control	88.5 ± 26.2	–	2.9 ± 0.6	–	3.3 ± 0.39	–	63.2 ± 8.9	–
Mealybug	96.5 ± 29.1	0.26	2.4 ± 0.6	0.59	5.2 ± 1.4	0.24	60.7 ± 12.8	0.61
JA	14.5 ± 5.9	0.004*	1.4 ± 0.5	0.08	3.8 ± 0.7	0.56	41.4 ± 8.5	0.09
SA	111.9 ± 122.6	0.53	4.4 ± 0.6	0.04*	4.6 ± 0.8	0.24	84.9 ± 6.3	0.03*

EPGs were recorded for 12 h per insect. Control: undamaged plant (n = 15); Mealybug: plant pre-infested with 40 adults of *P. solenopsis* for 120 h (n = 10); JA: plant pre-treated with 1.0 mL/leaf of JA solution for 24 h (n = 10); SA: plant pre-treated with 1.0 mL/leaf of SA solution for 24 h (n = 16). Values are means ± SE. Potential drop: caused by stylets puncturing cells; SEP: sieve element phase, observed during feeding in a sieve element. Rate of SEP feeding: the number of probes in sieve element phase during 12-h recording period.

*indicates significant difference (at α = 0.05) from control treatment as determined by one-way ANOVA.
